# Deep forest

**DOI:** 10.1093/nsr/nwy108

**Published:** 2018-10-08

**Authors:** Zhi-Hua Zhou, Ji Feng

**Affiliations:** National Key Laboratory for Novel Software Technology, Nanjing University, Nanjing 210023, China

**Keywords:** deep forest, deep learning, machine learning, ensemble methods, decision trees

## Abstract

Current deep-learning models are mostly built upon neural networks, i.e. multiple layers of parameterized differentiable non-linear modules that can be trained by backpropagation. In this paper, we explore the possibility of building deep models based on non-differentiable modules such as decision trees. After a discussion about the mystery behind deep neural networks, particularly by contrasting them with shallow neural networks and traditional machine-learning techniques such as decision trees and boosting machines, we conjecture that the success of deep neural networks owes much to three characteristics, i.e. layer-by-layer processing, in-model feature transformation and sufficient model complexity. On one hand, our conjecture may offer inspiration for theoretical understanding of deep learning; on the other hand, to verify the conjecture, we propose an approach that generates deep forest holding these characteristics. This is a decision-tree ensemble approach, with fewer hyper-parameters than deep neural networks, and its model complexity can be automatically determined in a data-dependent way. Experiments show that its performance is quite robust to hyper-parameter settings, such that in most cases, even across different data from different domains, it is able to achieve excellent performance by using the same default setting. This study opens the door to deep learning based on non-differentiable modules without gradient-based adjustment, and exhibits the possibility of constructing deep models without backpropagation.

## Introduction

Deep learning [1] has become a hot topic in various domains. However, what is deep learning? Answers from the crowd are very likely to be that ‘deep learning is a subfield of machine learning that uses deep neural networks' [2]. Actually, the great success of deep neural networks (DNNs) in tasks involving visual and audio information led to the rise of deep learning, and almost all current deep-learning applications are built upon neural network models or, more technically, multiple layers of parameterized differentiable non-linear modules that can be trained by backpropagation.

Though deep neural networks are powerful, they have many deficiencies. First, DNNs have too many hyper-parameters, and the learning performance depends seriously on careful parameter tuning. Indeed, even when several authors all use convolutional neural networks [3–5], they are actually using different learning models due to the many different options such as convolutional layer structures. This fact makes the training of DNNs very tricky, and theoretical analysis of DNNs extremely difficult because of too many interfering factors with almost infinite configurational combinations. Second, it is well known that the training of DNNs requires a huge amount of training data, and thus DNNs can hardly be applied to tasks where only small-scale training data are available. Note that even in the big data era, many real tasks still have an insufficient amount of training data due to the high cost of labeling, leading to inferior performance of DNNs in these tasks. Third, the network architecture has to be determined before training, and thus the model complexity is determined in advance. Actually, deep models are usually more complicated than necessary, as verified by the observation that recently there have been many reports about DNN performance improvement by adding shortcut connections [6,7], pruning [8,9], binarization [10,11], etc., as these operations simplify the original networks and actually decrease model complexity. It might be better if the model complexity could be determined automatically in a data-dependent way. Furthermore, it is well known that neural networks are black-box models whose decision processes are hard to explain. It is also noteworthy that, although DNNs have been well developed, there are still many tasks on which DNNs are inferior; for example, random forest [12] or XGBoost [13] are winners on many Kaggle competition tasks.

In order to tackle complicated learning tasks, learning models are likely to have to go deep. Current deep models, however, are always built upon neural networks. As discussed above, there are good reasons to explore non-NN-style deep models or, in other words, to consider whether deep learning can be realized with other modules, as they have their own advantages and may exhibit great potential if they are able to go deep. In particular, considering that neural networks are multiple layers of parameterized differentiable non-linear modules, whereas not all properties in the world are differentiable or best modeled as differentiable, in this paper we attempt to address the question of whether deep learning can be realized with non-differentiable modules.

The answer to the question may help understand important issues such as (1) do deep models have to be DNNs, or must deep models be constructed with differentiable modules? Note that there is research involving non-differentiable activation functions in DNNs; however, it usually uses differentiable functions that give an upper bound to the non-differentiable ones for relaxation in the optimization/learning process, and thus they actually still work with differentiable modules. (2) Is it possible to train deep models without backpropagation? Note that backpropagation and gradient-based adjustment require differentiability. (3) Is it possible to enable deep models to win tasks on which other models such as random forest or XGBoost are now superior? Actually, the machine-learning community has developed lots of learning modules, but many of them are non-differentiable and can hardly be tuned by gradient-based adjustment; it would be interesting to know whether it is possible to construct deep models based on these modules.

In this paper, we extend our preliminary research [14], which proposes the gcForest (multi-grained cascade forest) approach for constructing deep forest, a non-NN-style deep model. This is a novel decision-tree ensemble, with a cascade structure that enables representation learning by forest. Its representational learning ability can be further enhanced by multi-grained scanning, potentially enabling gcForest to be contextually or structurally aware. The cascade levels can be automatically determined such that the model complexity can be determined in a data-dependent way; this enables gcForest to work well even with small-scale data, and enables users to control training costs according to the computational resources available. gcForest has much fewer hyper-parameters than DNNs do, and its performance is quite robust to hyper-parameter settings; our experiments show that in most cases, it is able to get excellent performance by using the default setting, even across different data from different domains.

The section entitled ‘Ensemble learning and diversity' briefly introduces ensemble learning and ‘What is crucial for deep models?' explains our design motivations by analyzing why deep learning works. The section entitled ‘The gcForest approach' proposes our approach, followed by experiments reported in the section entitled ‘Experiments'. The section entitled ‘Real application' briefly presents a real application and the following section discusses some related work. The section entitled ‘Future issues' raises some issues for future exploration, followed by a section with some concluding remarks.

## Ensemble learning and diversity

Ensemble learning [15] is a machine-learning paradigm where multiple learners (e.g. classifiers) are trained and combined for a task. It is well known that an ensemble can usually achieve better generalization performance than single learners.

To construct a good ensemble, the individual learners should be accurate and diverse. Combining only accurate learners is often inferior to combining some accurate learners with some relatively weaker ones, because the complementarity is more important than pure accuracy. Actually, a beautiful equation has been theoretically derived from error-ambiguity decomposition [16]:

(1) \begin{equation*} E = \bar{E} - \bar{A} \ , \end{equation*}

where *E* denotes the error of an ensemble, $\bar{E}$ denotes the average error of individual classifiers in the ensemble, and $\bar{A}$ denotes the average ambiguity, later called diversity, among the individual classifiers. This offers general guidance for ensemble construction; however, it cannot be taken as an objective function for optimization, because the ambiguity is mathematically defined in the derivation and cannot be operated directly. Later, the ensemble community designed many diversity measures, but none has been well accepted as the right definition for diversity [15], and ‘what is diversity?' remains the holy grail problem in this area.

In practice, diversity enhancement is based on randomness injection during training. Roughly speaking, there are four major categories of strategies [15]. The first is data sample manipulation, which works by generating different data samples for different individuals, e.g. bootstrap sampling for bagging [17] and sequential importance sampling for AdaBoost [18]. The second is input feature manipulation, which works by generating different feature subspaces for different individuals, e.g. random subspace [19] randomly picks different subsets of features for different individuals. The third is learning parameter manipulation, which works by using different parameter settings of the base learning algorithm to generate different individuals, e.g. different initial weights can be used for different individual neural networks. The fourth is output representation manipulation, which works by using different output representations for different individuals, e.g. ECOC [20] employs error-correcting output codes, whereas flipping output [21] randomly switches labels of training instances. Different strategies can be used together. No strategy is always effective, e.g. data sample manipulation does not work well with stable learners whose performance does not significantly change according to slight modification of training data. More information about ensemble learning can be found in [15]. Our gcForest can be viewed as a decision-tree ensemble approach that utilizes almost all categories of strategies for diversity enhancement.

## What is crucial for deep models?

**Figure 1.**
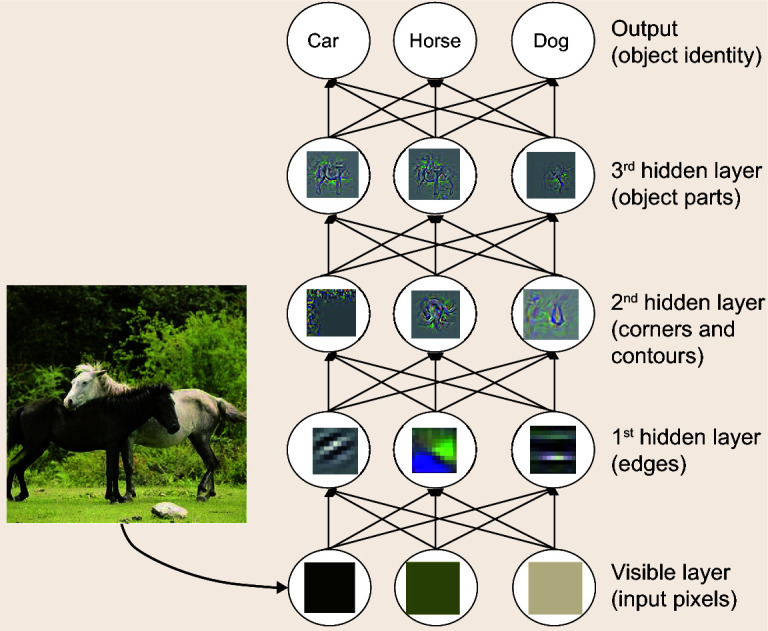
Illustration of the layer-by-layer processing in deep neural networks: Features of higher levels of abstract emerge as the layers build up.

It is widely recognized that the representation learning ability is crucial for the success of deep neural networks. What is crucial for representation learning in DNNs? We believe that the answer is layer-by-layer processing. Figure 1 provides an illustration simulated from a figure in [1], where features of higher levels of abstract emerge as the layers build up.

Considering that, if other issues are fixed, large model complexity (or more accurately, model capacity) generally leads to strong learning ability, maybe it sounds reasonable to attribute the successes of DNNs to the huge model complexity. This, however, cannot explain the fact that shallow networks are not as successful as deep ones, as one can increase the complexity of shallow networks to almost arbitrarily high by adding a nearly infinite number of hidden units. Consequently, we believe that the model complexity itself cannot explain the success of DNNs. Instead, we conjecture that the layer-by-layer processing is one of the most important factors behind DNNs because shallow networks, no matter how large their complexity can be, cannot do layer-by-layer processing. This conjecture offers important inspiration for the design of gcForest.

Then, how do we explain the fact that traditional learning models that can do layer-by-layer processing, e.g. decision trees and boosting machines, are not as successful as DNNs? We believe that the distinguishing factor lies in the fact that, in contrast to DNNs where new features are generated as illustrated in Fig. 1, decision trees and boosting machines always work on the original feature representation during the learning process; in other words, there is no in-model feature transformation. Moreover, in contrast to DNNs that can be endowed with arbitrarily high model complexity, decision trees and boosting machines can only have limited model complexity. Though model complexity itself does not give rise to the successes of DNNs, it is still important because large model capacity is needed if one wants to exploit big training data.

Overall, we conjecture that behind the mystery of DNNs there are three crucial characteristics, i.e. layer-by-layer processing, in-model feature transformation, and sufficient model complexity. To verify our conjecture, in the next section we will try to endow non-NN-style deep models with these characteristics.

## The gcForest approach

### Cascade forest structure

**Figure 2.**
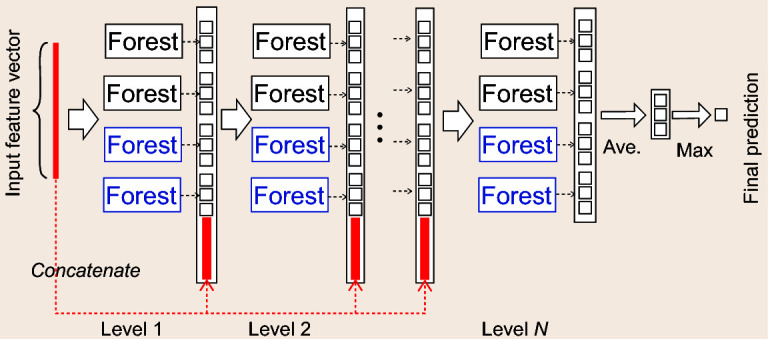
Illustration of the cascade forest structure. Suppose that each level of the cascade consists of two random forests (black) and two completely random forests (blue). Suppose that there are three classes to predict; thus, each forest will output a 3D class vector, which is then concatenated for re-representation of the input.

To realize layer-by-layer processing, gcForest employs a cascade structure, as illustrated in Fig. 2, where each level of the cascade receives feature information processed by the preceding level.

Each level is an ensemble of decision-tree forests, i.e. an ensemble of ensembles. Here, we include different types of forests to encourage diversity. For simplicity, suppose that we use two completely random forests and two random forests [12]. Each completely random forest contains 500 completely random trees [22], generated by randomly assigning a feature for splitting at each node, and growing a tree till pure leaf, i.e. each leaf node contains only the same class of instances. Similarly, each random forest contains 500 trees, by randomly picking $\sqrt{d}$ number of features as candidates (*d* is the number of input features) and selecting the one with the best Gini value for splitting. The number of trees in each forest is a hyper-parameter.

**Figure 3.**
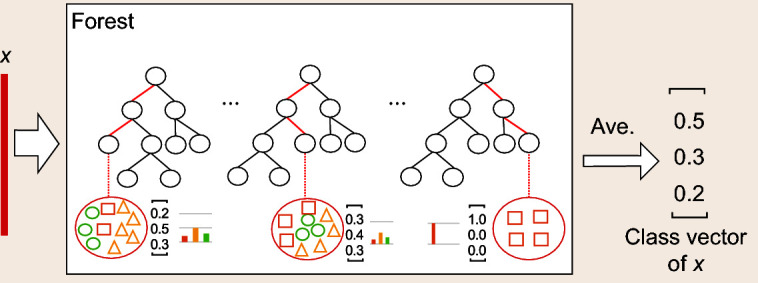
Illustration of class vector generation. Different marks in leaf nodes imply different classes; red highlights paths along which the concerned instance traverses to leaf nodes.

Given an instance, each forest can produce an estimate of class distribution, by counting the percentage of different classes of training examples at the leaf node where the concerned instance falls, and then averaging across all trees in the same forest, as illustrated in Fig. 3.

The estimated class distribution forms a class vector, which is then concatenated with the original feature vector to input to the next level. For example, suppose there are three classes; each of the four forests will produce a 3D class vector; consequently, the next level will receive 12 (= 3 × 4) augmented features.

Note that here we take the simplest form of class vectors, i.e. the class distribution at the leaf nodes into which the concerned instance falls. It is evident that such a small number of augmented features may deliver very limited augmented information, and it is very likely to be drowned out when the original feature vectors are high-dimensional. We will show in experiments that such a simple feature augmentation is already beneficial, and it is expected that more profit can be obtained if more augmented features are involved. Actually, it is apparent that more features may be incorporated, such as class distribution of the parent nodes, which express prior distribution, the sibling nodes, which express complementary distribution, etc. We leave these possibilities for future exploration.

To reduce the risk of overfitting, the class vector produced by each forest is generated by *k*-fold cross validation. In detail, each instance will be used as training data *k* − 1 times, resulting in *k* − 1 class vectors, which are then averaged to produce the final class vector as augmented features for the next level of the cascade. After expanding a new level, the performance of the whole cascade can be estimated on the validation set, and the training procedure will terminate if there is no significant performance gain; thus, the number of cascade levels can be automatically determined. Note that the training error rather than cross-validation error can also be used to control the cascade growth when the training cost is concerned or limited computation resources are available. In contrast to most deep neural networks whose model complexity is fixed, gcForest adaptively decides its model complexity by terminating training when adequate. This enables it to be applicable to different scales of training data, not limited to large-scale ones.

### Multi-grained scanning

Deep neural networks are powerful in handling feature relationships, e.g. convolutional neural networks are effective on image data where spatial relationships among the raw pixels are critical; recurrent neural networks are effective on sequence data where sequential relationships are critical. Inspired by this recognition, we enhance the cascade forest with a procedure of multi-grained scanning.

**Figure 4.**
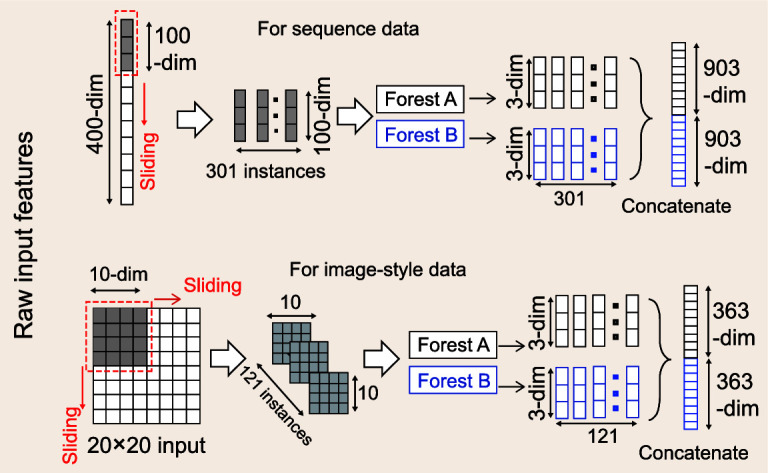
Illustration of feature re-representation using sliding window scanning. Suppose that there are three classes, the raw features are 400-dim, and the sliding window is 100-dim.

As Fig. 4 illustrates, sliding windows are used to scan the raw features. Suppose that there are 400 raw features and a window size of 100 features is used. For sequence data, a 100D feature vector will be generated by sliding the window for one feature; in total 301 feature vectors are produced. If the raw features have spatial relationships, such as a 20 × 20 panel of 400 image pixels, then a 10 × 10 window will produce 121 feature vectors (i.e. 121 10 × 10 panels). All feature vectors extracted from positive/negative training examples are regarded as positive/negative instances, which will then be used to generate class vectors as in the section entitled ‘Cascade forest structure': instances extracted from the same size of windows will be used to train a completely random forest and a random forest, and then the class vectors are generated and concatenated as transformed features. As illustrated in Fig. 4, for three classes, 301 3D class vectors are produced by each forest, leading to a 1806D transformed feature vector corresponding to each 400D raw feature vector.

For instances extracted from the windows, we simply assign them with the label of the original training example. Some label assignments are inherently incorrect. For example, suppose that the original training example is a positive image about ‘car'; it is clear that many extracted instances do not contain a car, and therefore they are incorrectly labeled as positive. This is actually related to flipping output [21], an approach for ensemble diversity enhancement.

Note that when transformed feature vectors are too long to be accommodated, feature sampling can be performed, e.g. by subsampling the instances generated by sliding window scanning, as completely random trees do not rely on feature split selection whereas random forests are quite insensitive to feature split selection. The feature sampling process is also related to random subspace [19], an approach for ensemble diversity enhancement.

**Figure 5.**
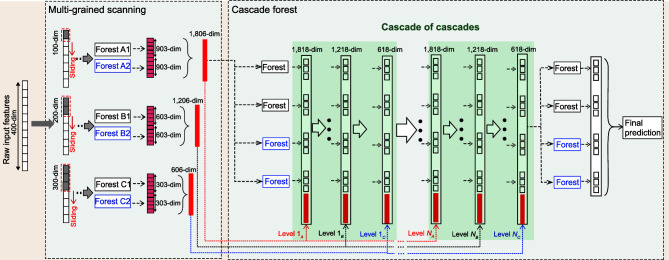
Overall procedure of gcForest. Suppose that there are three classes, the raw features are 400-dim, and three sizes of sliding windows are used.

Figure 4 shows only one size of sliding window. By using multiple sizes of sliding windows, multi-grained feature vectors will be generated, as shown in Fig. 5.

### Overall procedure and hyper-parameters

Figure 5 summarizes the overall procedure of gcForest. Suppose that the original input is of 400 raw features, and three window sizes are used for multi-grained scanning. For *m* training examples, a window with a size of 100 features will generate a data set of 301 × *m* 100D training examples. These data will be used to train a completely random forest and a random forest, each containing 500 trees. If there are three classes to be predicted, a 1806D feature vector will be obtained as described in the section entitled ‘Cascade forest structure'. The transformed training set will then be used to train the first grade of the cascade forest.

Similarly, sliding windows with sizes of 200 and 300 features will generate 1206D and 606D feature vectors, respectively, for each original training example. The transformed feature vectors, augmented with the class vector generated by the previous grade, will then be used to train the second and third grades of the cascade forests, respectively. This procedure will be repeated till convergence of validation performance. In other words, the final model is actually a cascade of cascades, where each cascade consists of multiple levels each corresponding to a grain of scanning, e.g. the first cascade consists of Level 1_*A*_ to Level 1_*C*_ in Fig. 5. For difficult tasks, more grains can be used when computational resources allow.

Given a test instance, the multi-grained scanning procedure will be gone through to get the corresponding transformed feature representation, and then the cascade will be gone through till the last level. The final prediction will be obtained by aggregating the four 3D class vectors at the last level, and the class with the maximum aggregated value will be output.

Table 1 summarizes the hyper-parameters of typical DNNs and gcForest, where the default values of gcForest used in our experiments are given.

**Table 1.** Summary of hyper-parameters and default settings of gcForest. Bold font highlights hyper-parameters with relatively larger influence; ‘?' indicates default value unknown, or a general requirement for different settings for different tasks.

Deep neural networks (e.g. convolutional neural networks)	gcForest
Type of activation functions:	Type of forests:
Sigmoid, ReLU, tanh, linear, etc.	Completely random forest, random forest, etc.
Architecture configurations:	Forest in multi-grained scanning:
**No. hidden layers**: ?	**No. forests**: {2}
**No. nodes in hidden layer**: ?	**No. trees in each forest**: {500}
**No. feature maps**: ?	Tree growth: till pure leaf, or reach depth 100
**Kernel size**: ?	**Sliding window size**: {⌊*d*/16⌋, ⌊*d*/8⌋, ⌊*d*/4⌋}
Optimization configurations:	Forest in cascade:
**Learning rate**: ?	**No. forests**: {8}
Dropout: {0.25/0.50}	**No. trees in each forest**: {500}
**Momentum**: ?	Tree growth: till pure leaf
**L1/L2 weight regularization penalty**: ?	
Weight initialization: Uniform, glorot_normal, glorot_uni, etc.	
Batch size: {32/64/128}	

## Experiments

### Configuration

We compare gcForest with deep neural networks and several other popular learning algorithms. The implementations are based on Python, with neural networks from Keras and traditional learning algorithms from Sklearn.

In all experiments gcForest is using the same cascade structure: Each level consists of four completely random forests and four random forests, each containing 500 trees, as described in the section entitled ‘Cascade forest structure'. Three-fold cross validation is used for class vector generation. The number of cascade levels is automatically determined. In detail, we split the training set into two parts, i.e. a growing set and an estimating set. (Some experimental datasets are given with training/validation sets. To avoid confusion, here we call the subsets generated from the training set the growing/estimating sets.) Then we use the growing set to grow the cascade, and the estimating set to estimate the performance. If growing a new level does not improve the performance, the growth of the cascade terminates and the estimated number of levels is obtained. Then, the cascade is retrained based on merging the growing and estimating sets. For all experiments we take 80% of the training data for the growing set and 20% for the estimating set. For multi-grained scanning, three window sizes are used. For *d* raw features, we use feature windows with sizes of ⌊*d*/16⌋, ⌊*d*/8⌋, ⌊*d*/4⌋; if the raw features have panel structure (such as images), the feature windows also have panel structure, as shown in Fig. 4. Note that a careful task-specific tuning will lead to better performance; here, to highlight that the hyper-parameter setting of gcForest is much easier than deep neural networks, we simply use the same setting for all tasks, whereas task-specific tunings are conducted for DNNs.

For deep neural network configurations, we use ReLU for the activation function, cross-entropy for the loss function, Adadelta for optimization, and a dropout rate of 0.25 or 0.5 for hidden layers according to the scale of the training data. The network structure hyper-parameters, however, cannot be fixed across tasks, otherwise the performance will be embarrassingly unsatisfactory. For example, a network attained 80% accuracy on the ADULT dataset but achieved only 30% accuracy on YEAST with the same architecture (only the numbers of input/output nodes were changed to suit the data). Therefore, for DNNs, we examine a variety of architectures on the validation set and pick the one with the best performance, then re-train the network on the training set and report the test accuracy.

### Results

We run experiments on a broad range of tasks, with data types of image, audio, time series, text, etc.

#### Image categorization

The MNIST dataset [3] contains 60 000 images of size 28 × 28 for training (and validating), and 10 000 images for testing. Deep belief nets in [23] attained an accuracy of 98.75%, a re-implementation of LeNet-5 (a modern version of LeNet with dropout and ReLUs) attained an accuracy of 99.05%, SVM (rbf kernel) 98.60%, and random forest 96.80%, whereas gcForest attains 99.26% by simply using the default settings in Table 1.

#### Face recognition

The ORL dataset [24] contains 400 grayscale facial images taken from 40 persons. We randomly choose five/seven/nine images per person for training, and report the test performance on the remaining images. Note that a random guess will achieve 2.5% accuracy, since there are 40 possible outcomes. We compare with a CNN consisting of two convolutional layers (conv-layers) with 32 feature maps of 3 × 3 kernel, and each conv-layer has a 2 × 2 max-pooling layer following it. A dense layer of 128 hidden units is fully connected with the convolutional layers and finally a fully connected soft-max layer with 40 hidden units is appended at the end. ReLU, cross-entropy loss, a dropout rate of 0.25 and Adadelta are used for training. The batch size is set to 10, and 50 epochs are used. We have also tried other configurations of CNN, but this one gives the best performance: test accuracies of 86.50%/91.67%/95.00%, corresponding to five/seven/nine training images per person. The *k*NN (*k* = 3) test accuracies are 76.00%/83.33%/92.50%, SVM (rbf kernel) 80.50%/82.50%/85.00%, and random forest 91.00%/93.33%/95.00%, whereas gcForest attains 91.00%/96.67%/97.50% by simply using the default settings.

#### Music classification

The GTZAN dataset [25] contains 10 genres of music clips, each represented by 100 tracks 30 seconds long. We split the dataset into 700 clips for training and 300 clips for testing. In addition, we use the MFCC feature to represent each 30-second music clip, which transforms the original sound wave into a 1280 × 13 feature matrix. Each frame is atomic according to its own nature; thus, CNN uses a 13 × 8 kernel with 32 feature maps as the conv-layer, each followed by a pooling layer. Two fully connected layers with 1024 and 512 units, respectively, are appended, and finally a soft-max layer is added at the end. We also compare it with an MLP with two hidden layers, with 1024 and 512 units, respectively. Both networks use ReLU as the activation function and categorical cross-entropy as the loss function. For random forest, logistic regression and SVM, each input is concatenated into a 1280 × 13 feature vector. The test accuracies are: CNN 59.20%, MLP 58.00%, random forest 50.33%, logistic regression 50.00%, and SVM (rbf kernel) 18.33%, whereas gcForest attains 65.67% by simply using the default settings.

#### Hand movement recognition

The sEMG dataset [26] consists of 1800 records each belonging to one of six hand movements, i.e. spherical, tip, palmar, lateral, cylindrical and hook. This is a time-series dataset, where EMG sensors capture 500 features per second and each record is associated with 3000 features. In addition to an MLP with input–1024–512–output structure, we also evaluate a recurrent neural network, LSTM [27], with 128 hidden units and a sequence length of 6 (500-dim input vector per second). The test accuracies are: LSTM 45.37%, MLP 38.52%, random forest 29.62%, SVM (rbf kernel) 29.62%, and logistic regression 23.33%, whereas gcForest attains 71.30% by simply using the default settings.

#### Sentiment classification

The IMDB dataset [28] contains 25 000 movie reviews for training and 25 000 for testing. The reviews are text data represented by tf-idf features. These are not image data, and thus CNNs are not directly applicable; CNNs facilitated with word embedding achieved a test accuracy of 89.02% [29]. An MLP with the structure input–1024–1024–512–256–output attains 88.04%, SVM (rbf kernel) 87.56%, random forest 85.32%, and logistic regression 88.62%. Considering that tf-idf features do not convey spatial or sequential relationships, we use the default setting for gcForest but skip multi-grained scanning, and achieve a test accuracy of 89.16%, even better than CNN facilitated with word embedding.

#### Low-dimensional data

We also evaluate gcForest on UCI datasets [30] with a relatively small number of features: LETTER with 16 features and 16 000/4000 training/test examples, ADULT with 14 features and 32 561/16 281 training/test examples, and YEAST with only eight features and 1038/446 training/test examples. Fancy architectures like CNNs could not work on such data as there are too few features without spatial relationships. So, we compare with MLPs. Unfortunately, although MLPs have fewer configuration options than CNNs, they are still very tricky to set up. For example, an MLP with input–16–8–8–output structure and ReLU activation achieves 76.37% accuracy on ADULT but just 33% on LETTER. We conclude that there is no way to have one MLP structure that gives decent performance across all datasets. Therefore, we report different MLP structures with the best performance: for LETTER the structure is input–70–50–output with test accuracy 95.70%, for ADULT it is input–30–20–output with test accuracy 85.25%, and for YEAST it is input–50–30–output with test accuracy 55.60%. These results are inferior to random forest: 96.50% on LETTER, 85.49% on ADULT, 61.66% on YEAST. In contrast, gcForest achieves 97.40% on LETTER, 86.40% on ADULT, 63.45% on YEAST, by simply using the default setting and abandoning multi-grained scanning by considering that the features of these small-scale data do not hold spatial/sequential relationships.

#### High-dimensional data

The CIFAR-10 dataset [31] contains 50 000 images of 10 classes for training and 10 000 images for testing. Here, each image is a 32 × 32 colored image with eight gray-levels; thus, each instance is of 8192-dim. ResNet achieved test accuracy 93.57% [7], AlexNet 83.00% [4], deep belief net 62.20% [31], and MLP 42.20% [32]. The test accuracies of non-DNN approaches are: random forest 50.17%, logistic regression 37.32%, and SVM (linear kernel) 16.32%.

As we discussed in the section entitled ‘The gcForest approach', currently we include only a 10-dim augmented feature vector from each forest, and such a small number of augmented features will be easily drowned out by the original long feature vector. Nevertheless, though the test accuracy of gcForest with the default setting, 61.78%, is inferior to state-of-the-art DNNs, it is already the best among non-DNN approaches. Moreover, the performance of gcForest can be further improved via task-specific tuning, e.g. by including more grains (i.e. using more sliding window sizes in multi-grained scanning) like gcForest(5grains), which uses five grains and attains 63.37%. It is also interesting to see that the performance undergoes a significant improvement to 69.00% with gcForest(gbdt), which simply replaces the final level with GBDT [13]. The section entitled ‘Influence of larger models' will show that better performance can be obtained if larger models of gcForest can be trained.

### Running time

Our experiments use a PC with two Intel E5 2695 v4 CPUs (18 cores), and the running efficiency of gcForest is good. For example, for the IMDB dataset (25 000 examples with 5000 features), it takes 267.1 seconds per cascade level, and automatically terminates with nine cascade levels, amounting to 2404 seconds or 40 minutes. In contrast, an MLP compared on the same dataset requires 50 epochs for convergence and 93 seconds per epoch, amounting to 4650 seconds or 77.5 minutes for training; 14 seconds per epoch (with a batch size of 32) if using a GPU (Nvidia Titan X pascal), amounting to 700 seconds or 11.6 minutes. Multi-grained scanning will increase the cost of gcForest; however, the different grains of scanning are inherently parallel. Also, both completely random forest and random forest are parallel ensemble methods [15]. Thus, the efficiency of gcForest can be improved further with optimized parallel implementation. Note that the training cost is controllable because users can set the number of grains, forests, and trees by considering the computational cost available. It is also noteworthy that the above comparison is somewhat unfair to gcForest, because many different architectures have been tried for neural networks to achieve reported performance but these time costs are not included.

### Performance tendency

**Figure 6.**
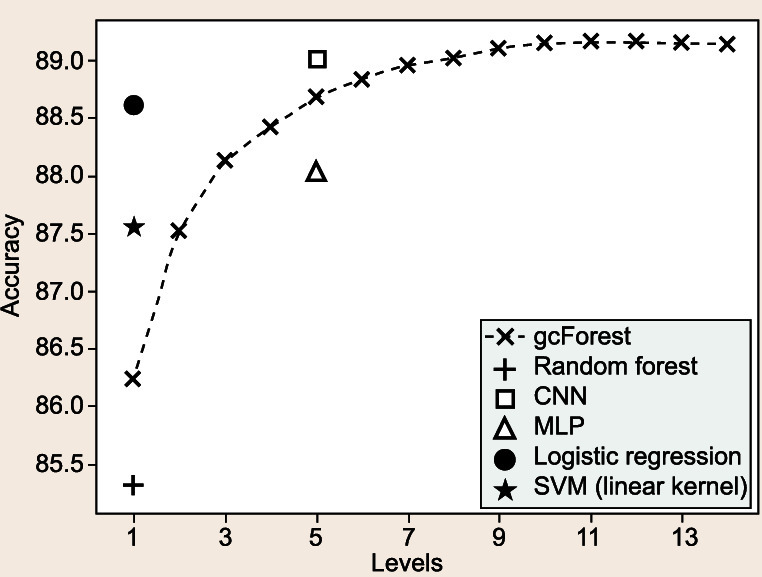
Performance tendency on IMDB.

Figure 6 shows the performance tendency of gcForest when the number of cascade levels increases. It can be seen that gcForest starts with a performance inferior to SVM and MLP, and gradually improves. In our experiments the validation process terminates the growth at the ninth level; the figure shows more levels for observing the tendency.

### Influence of multi-grained scanning

To study the separate contribution of the cascade forest structure and multi-grained scanning, we compare gcForest with a cascade forest on the MNIST, GTZAN and sEMG datasets. The test accuracies with/without multi-grained scanning are 99.26%/65.67%/71.30% and 98.02%/52.33%/48.15% on MNIST/GTZAN/sEMG, respectively. It is evident that, when there are spatial or sequential feature relationships, multi-grained scanning helps improve performance.

### Influence of completely random forest

To study the contribution of completely random forest, we compare gcForest with its variant, which replaces completely random forests by random forests. The test accuracies with/without completely random forests are 99.26%/89.16%/97.40% and 99.11%/87.62%/96.65% on MNIST/IMDB/LETTER, respectively. It shows that completely random forest helps no matter whether multi-grained scanning is applied (MNIST) or not (IMDB), or whether data are low-dimensional (LETTER).

### Influence of cascade structure

**Figure 7.**
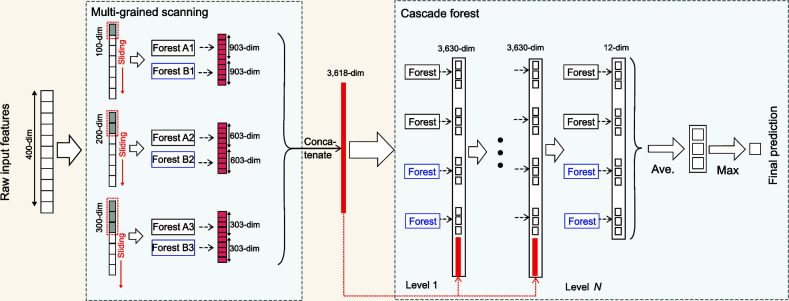
A variant that concatenates all features from multiple grains. Suppose that there are three classes, the raw features are 400-dim, and three sizes of sliding windows are used.

The final model structure of gcForest is a cascade of cascades, where each cascade consists of multiple levels each corresponding to a grain of scanning, as shown in Fig. 5. There are other possible ways to exploit the features from multiple grains, e.g. the variant that concatenates all features together as shown in Fig. 7.

Table 2 shows that concatenating the features from multiple grains is not as good as the current design in gcForest (here, ORL has nine training images per person). Nevertheless, there might be other ways leading to better results; we leave this for future exploration.

**Table 2.** Results with the variant of concatenating features from multiple grains.

	MNIST	ORL	GTZAN	sEMG
gcForest	**99.26%**	97.50%	65.67%	**71.30%**
variant	98.96%	**98.30%**	65.67%	55.93%
	IMDB	LETTER	ADULT	YEAST
gcForest	89.16%	**97.40%**	**86.40%**	**63.45%**
variant	**89.32%**	97.25%	86.17%	63.23%

### Influence of larger models

**Figure 8.**
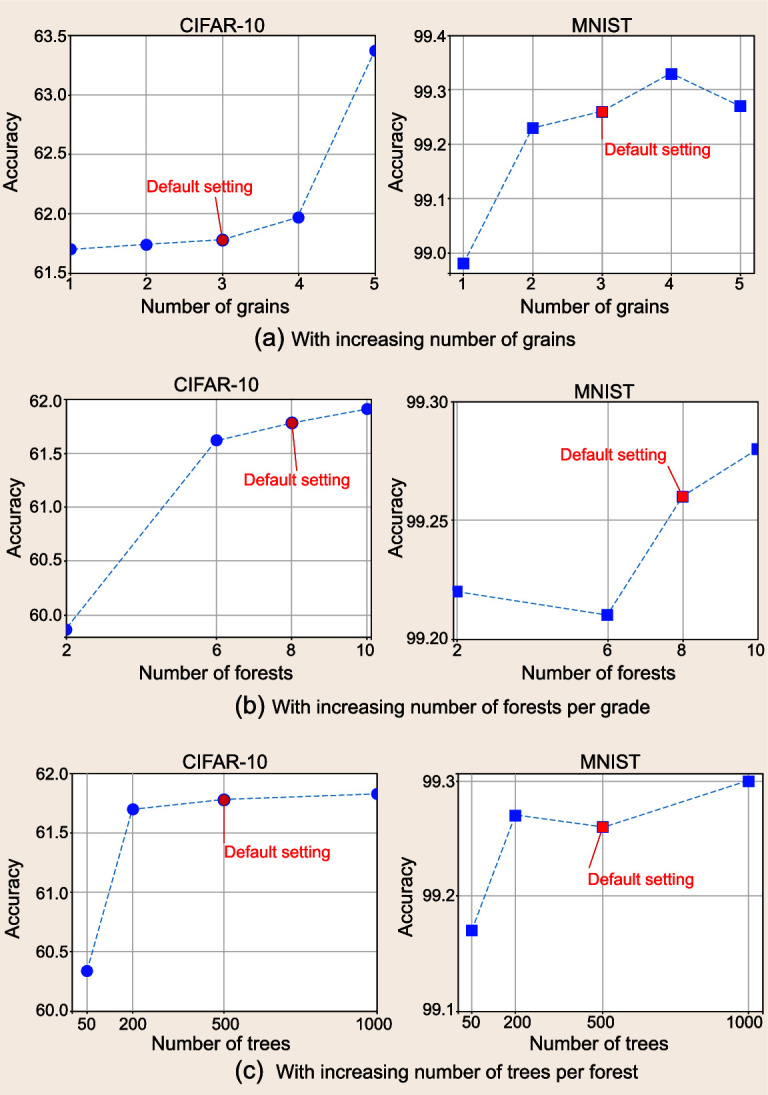
Performance with increasing number of grains/forests/trees. Red highlights the performance with the default setting.

Figure 8 suggests that larger models tend to offer better performance, though we have not tried even more grains, forests and trees due to the limits of computational resources.

Note that computational facilities are crucial for enabling the training of larger models; e.g. GPUs for DNNs. On one hand, some new computational devices, such as Intel KNL of the MIC (many integrated core) architecture, may offer acceleration for gcForest. On the other hand, some components of gcForest, e.g. multi-grained scanning, may be accelerated by GPUs. Moreover, there is plenty of room for improvement with distributed computing implementations.

## Real application

gcForest has been implemented in an industrial distributed-machine-learning platform and applied to real-world illegal cash-out fraud detection by a big unicorn enterprise [33]. On a dataset with 131 407 704 training examples and 52 489 529 testing examples, each corresponding to a transaction described by 5000 features, gcForest achieved the best performance of 0.9970/0.5440/0.9480 on AUC/F1/KS, whereas DNNs achieved 0.9722/0.3861/0.8551. For details see [33].

## Related work

The gcForest is a decision-tree ensemble approach. There are studies showing that by using ensembles such as random forest facilitated with DNN features, the performance can be even better than simply using DNNs [34]. Our purpose is quite different. We are aiming at a non-NN-style deep model rather than a combination with DNNs. By using cascade forest structure, we hope to endow the model with characteristics of layer-by-layer processing, in-model feature transformation and sufficient model complexity.

Random forest [12], which has been widely applied to various tasks, is one of the most successful ensemble methods. Completely random forest has been found useful during recent years, such as iForest [35] for anomaly detection, sencForest [36] for handling emerging new classes in streaming data, etc. gcForest offers another example exhibiting the usefulness of completely random forest.

Many works have tried to connect random forest with neural networks, such as converting cascaded random forest to CNNs [37] and exploiting random forest to help initialize neural networks [38]. These works are typically based on early studies, e.g. mapping of trees to networks, tree-structured neural networks, as reviewed in [39]. Their goals are totally different from ours. In particular, their final models are based on differentiable modules (even for studies involving non-differentiable activation functions, differentiable relaxation functions are actually used in the optimization/learning process), whereas we are trying to develop deep models based on non-differentiable modules without relying on gradient-based adjustment.

The multi-grained scanning procedure of gcForest uses different sizes of sliding windows to examine the data; this is somewhat related to wavelet and other multi-resolution examination procedures [40]. For each window size, a set of instances is generated from one training example; this is related to bag generators of multi-instance learning [41]. In particular, the bottom part of Fig. 4, if applied to images, can be regarded as the SB image bag generator [41].

The cascade procedure of gcForest is related to boosting [18], which is able to automatically decide the number of learners in an ensemble, and in particular a cascade boosting procedure [42] has achieved great success in object detection tasks. Note that when multiple grains are used, each level in the cascade of gcForest consists of multiple grades; this is actually a cascade of cascades. Each grade can be regarded as an ensemble of ensembles.

Passing outputs of one level of learners as inputs to another level of learners is related to stacking [43,44]. However, stacking is easy to overfit with more than two levels, and can hardly enable a deep model by itself. Our trick lies in the enhancement of diversity during model growth. Actually, gcForest exploits all major categories of diversity enhancement strategies [15].

As a tree-based approach, gcForest might be potentially more useful for theoretical analysis than deep neural networks, although this is beyond the scope of this paper. Indeed, some recent theoretical studies about deep learning, e.g. [45], seem more intimate with tree-based models.

## Future issues

One important future issue is to enhance the feature re-representation process. The current implementation of gcForest takes the simplest form of class vectors, i.e. the class distribution at the leaf nodes into which the concerned instance falls. Such a small number of augmented features will be easily drowned out when the original feature vectors are high-dimensional. It is apparent that more features may be involved. Intuitively, more features may enable the incorporation of more information, although this is not always necessarily helpful for generalization. Moreover, a longer class vector may enable a joint multi-grained scanning process, leading to more flexibility of re-representation. Recently we have shown that a decision forest can serve as AutoEncoder [46]. On one hand, this shows that the ability of AutoEncoder is not a special property of neural networks; on the other hand, this shows that a forest can encode abundant information, offering great potential to facilitate feature re-representation.

Another important future issue is to accelerate and reduce the memory consumption. Building larger models may lead to better performance, where computational facilities are crucial for enabling the training of larger models. The success of DNNs owes much to the acceleration offered by GPUs, but forest structure is unfortunately not suitable for GPUs. One possibility is to consider some new computational devices such as KNL; another is distributed computing implementation. Feature sampling can be executed when transformed feature vectors produced by multi-grained scanning are too long to be accommodated; this not only helps storage reduction, but also offers another channel to enhance the diversity. It is also possible to explore smarter sampling strategies such as BLB [47] or feature hashing [48] when adequate. The hard negative mining strategy may help improve generalization, and efforts to improve the efficiency of hard negative mining may also be helpful for multi-grained scanning [49]. The efficiency of gcForest may be further improved by reusing some components during the process of different grained scanning, class vectors generation, forest training, completely random tree generation, etc. In case the learned model is big, it is possible to reduce it to a smaller one by using the strategy presented in [50], later called knowledge distillation.

The adoption of completely random forest not only helps diversity enhancement, but also provides an opportunity to exploit unlabeled data. Note that the growth of completely random trees does not require labels; label information is only needed for annotating leaf nodes. Intuitively, for each leaf node it might be possible to require only one labeled example if the node is to be annotated according to the majority cluster on the node, or one labeled example per cluster if all clusters in the node are non-negligible. This offers gcForest the opportunity of incorporating active learning [51] and/or semi-supervised learning strategies [52].

The gcForest is able to achieve a performance highly competitive with DNNs on a broad range of tasks except some large-scale image tasks. Indeed, DNNs are very successful in image tasks, e.g. [53,54]. On one hand, we believe that the performance of gcForest can be significantly improved, e.g. by designing a better feature re-representation scheme rather than using the current simple classification vectors. On the other hand, it should not be ignored that DNN models have been investigated for more than 20 years by a huge crowd of researchers/engineers whereas deep forest has just been born. Furthermore, we conjecture that numeric modeling tasks such as image/audio data are very suitable for DNNs because their operations, such as convolution, fit well with numeric signal modeling. Deep forest was not developed to replace DNNs for such tasks; instead, it offers an alternative when DNNs are not superior. There are plenty of tasks, especially categorical/symbolic or mixed modeling tasks, where deep forest may be found useful. For example, the application described in the section entitled ‘Real application' is a mixed modeling task involving both categorical and numeric features.

## Conclusion

This paper attempts to address the question of whether deep learning can be realized with non-differentiable modules. We conjecture that behind the mystery of deep neural networks there are three crucial characteristics, i.e. layer-by-layer processing, in-model feature transformation, and sufficient model complexity. To verify the conjecture, we try to endow a non-NN-style deep model with these characteristics, and our results show that it really works.

The proposed gcForest approach (a shared gcForest code for small- or medium-scale data is available at [55]) is able to construct deep forest, a deep model based on decision trees, and the training process does not rely on backpropagation and gradient adjustment. Compared with deep neural networks, gcForest has fewer hyper-parameters and has achieved excellent performance across various domains even by using the same parameter settings.

There are other possibilities for constructing deep forest. As a seminal study, we have only explored a little in this direction. Indeed, the most important value of this paper lies in the fact that it may open the door for non-NN-style deep learning, or deep models based on non-differentiable modules that do not rely on gradients.
